# Neural Stem Cells in the Adult Subventricular Zone Oxidize Fatty Acids to Produce Energy and Support Neurogenic Activity

**DOI:** 10.1002/stem.2042

**Published:** 2015-06-04

**Authors:** Elizabeth A. Stoll, Rebecca Makin, Ian R. Sweet, Andrew J. Trevelyan, Satomi Miwa, Philip J. Horner, Douglass M. Turnbull

**Affiliations:** ^1^ Centre for Brain Ageing and VitalityNewcastle UniversityNewcastle upon TyneUnited Kingdom; ^2^ Wellcome Trust Centre for Mitochondrial ResearchInstitute for Ageing and HealthNewcastle UniversityNewcastle upon TyneUnited Kingdom; ^3^ Institute for Ageing and HealthNewcastle UniversityNewcastle upon TyneUnited Kingdom; ^4^ Institute of NeuroscienceNewcastle UniversityNewcastle upon TyneUnited Kingdom; ^5^ Undergraduate Programme in Biomedical SciencesNewcastle UniversityNewcastle upon TyneUnited Kingdom; ^6^ Division of MetabolismEndocrinologyand NutritionUniversity of WashingtonSeattleUSA; ^7^ Institute for Stem Cell and Regenerative MedicineUniversity of WashingtonSeattleUSA

**Keywords:** Neural stem cell, Progenitor, Neurogenesis, Fatty acid oxidation, Proliferation, Differentiation

## Abstract

Neural activity is tightly coupled to energy consumption, particularly sugars such as glucose. However, we find that, unlike mature neurons and astrocytes, neural stem/progenitor cells (NSPCs) do not require glucose to sustain aerobic respiration. NSPCs within the adult subventricular zone (SVZ) express enzymes required for fatty acid oxidation and show sustained increases in oxygen consumption upon treatment with a polyunsaturated fatty acid. NSPCs also demonstrate sustained decreases in oxygen consumption upon treatment with etomoxir, an inhibitor of fatty acid oxidation. In addition, etomoxir decreases the proliferation of SVZ NSPCs without affecting cellular survival. Finally, higher levels of neurogenesis can be achieved in aged mice by ectopically expressing proliferator‐activated receptor gamma coactivator 1 alpha (PGC1α), a factor that increases cellular aerobic capacity by promoting mitochondrial biogenesis and metabolic gene transcription. Regulation of metabolic fuel availability could prove a powerful tool in promoting or limiting cellular proliferation in the central nervous system. Stem Cells *2015;33:2306–2319*

**Significance Statement**

Most cell types in the adult brain rely on sugars such as glucose to produce energy. In this study, we find that neural stem cells in the adult brain use fatty acids to power respiratory activity and cell division. The identification of metabolic substrates required by the adult neural stem cell is imperative to understanding the process of regeneration. Regulation of metabolic fuel availability may even prove a powerful tool in promoting or limiting cellular proliferation in the central nervous system.

## Introduction

Neural stem/progenitor cells (NSPCs), which retain the capacity to produce new neurons and glia in the adult mammalian brain, reside in the subventricular zone (SVZ) of the lateral ventricle and the subgranular zone of hippocampal dentate gyrus (DG) 1, 2. While newly born cells in the inner molecular cell layer of DG migrate locally, cells from SVZ migrate long distances along the rostral migratory stream to populate the olfactory bulb (OB) with new GABAergic interneurons including dopaminergic neurons 3. The control of neurogenesis in the adult brain is notoriously complex, and depends upon factors in both the extracellular environment and limitations within the cellular machinery 4. As NSPCs reside in a “neurogenic niche” fortified by blood vessels and astrocytes 5, these cells have direct access to contents in the bloodstream, including substrates to fuel energy metabolism. In this study, we investigated the substrates required by adult mammalian neural stem cells to maintain their metabolic and neurogenic activity.

Catabolism, the production of energy in the form of ATP, is necessary to sustain cellular activity. The adult brain primarily depends upon the oxidation of carbohydrates such as glucose to fuel energy production. Although the brain comprises about 2% of body weight, this organ accounts for approximately 25% of organismal glucose consumption and 20% of oxygen consumption. Neuronal activity is indeed tightly coupled to glucose uptake 6. However, intriguingly, neurons and astrocytes have recently been shown to compartmentalize metabolic processes 7, 8. Glutamate released by neurons during synaptic transmission triggers astrocytic uptake of blood‐borne glucose. Astrocytes then undergo glycolysis, metabolizing glucose into pyruvate then releasing excess end products in the form of lactate into the extracellular space via the monocarboxylate transporter 4 (MCT4) 9, 10. Neurons take up this extracellular lactate via the MCT2, and convert it back to pyruvate in a reaction catalyzed by lactate dehydrogenase 8, 11. Both neurons and astrocytes then use pyruvate intracellularly to fuel oxidative metabolism. Physiological studies over recent years have shown that neurons prefer lactate as an bioenergetic substrate 11, 12, although both glucose and lactate can strongly stimulate oxidative metabolism in mature neurons 12, 13. However, the metabolic fuel requirements of adult neural stem cells are not known.

In this report, we demonstrate that neural stem cells derived from the mouse SVZ do not require glucose to sustain oxygen consumption, unlike other cell types that have been characterized in the adult brain. Instead, adult NSPCs are capable of using multiple fuel sources to maintain the high levels of aerobic respiration necessary for cellular division. In particular, fatty acids stimulate respiratory activity in these cells.

Beta‐oxidation of fatty acids is performed by numerous cell types, most notably cardiac myocytes and skeletal muscle tissue 14. Although mature cells within the adult brain depend on carbohydrates such as glucose for energy production, the fetal brain oxidizes primarily free fatty acids and other related substrates derived from the mother's milk 15, 16. Metabolic dependence upon fatty acids appears to be retained in adult neural stem cells. In this study, we performed gain‐of‐function and loss‐of‐function studies to test whether beta‐oxidation contributes to energy production and acts as a limiting factor in cellular proliferation. Here, we report that adult neural stem cells in the SVZ rely upon fatty acid oxidation to support aerobic respiration and proliferative activity, although other fuels can also be oxidized.

## Materials and Methods

### Immunohistochemistry

All experiments were performed as approved by the University of Washington Institutional Animal Care and Use Committee (IACUC) and United Kingdom Home Office Project Licence Number 60/4386. Full details on experimental techniques are available in Supporting Information Materials and Methods. For immunohistochemical studies, wild‐type adult C57BL/6 mice were killed and brain tissues were processed for cryosectioning. Ten micrometer‐thick slices were subjected to 0.01 M sodium citrate at 100°C for 10 minutes for antigen retrieval. Sections were then rinsed with phosphate‐buffered saline (PBS) containing 0.1% Triton X‐100 (PBST). Nonspecific staining was blocked for 2 hours in PBS with 0.1% Triton X‐100 and 5% donkey serum (blocking solution). Sections were incubated overnight at 4°C with appropriate antibodies diluted in blocking solution (details in Supporting Information Table 1A). Sections were then rinsed with PBST. The appropriate secondary antibodies were diluted by 1:250 in blocking solution and placed on sections for 2 hours at room temperature (details in Supporting Information Table 1B). Sections were rinsed and costained with Hoechst diluted to 1 μg/ml in PBST. Fluorescence microscopy was performed using a Zeiss Apoptome Microscope with attached camera and Axiovision software.

### Laser‐Capture Microdissection

Cells within SVZ, OB, or cortex were microdissected using the Zeiss PALM MicroBeam Laser‐Capture Microdissection Microscope. Cells were collected using the Ambion RNAqueous MicroKit (Life Technologies, Carlsbad, CA, www.lifetechnologies.com) and stored at −20°C until ready for real‐time polymerase chain reaction (PCR) analysis.

### Real‐Time PCR

Real‐time PCR was used to quantify metabolic transcripts in laser‐captured tissue samples and serum‐exposed dissociated cells. The real‐time PCR was based on the procedure described previously 17, which quantifies fluorescence emitted by dyes VIC^®^ and FAM^®^ that are conjugated to probes complementary to cDNA reverse‐transcribed from mRNA transcripts. The assay was performed on the StepOnePlus Real‐Time PCR System (Life Technologies, Carlsbad, CA). Samples from each neuroanatomical area or serum‐exposure timepoint were each run in triplicate to quantify total copy number. Each sample was normalized to the standard curve, using the equation Value = (CT − Intercept)/Slope. These values were normalized to *B‐actin*. Total copy number of cDNA representing *ACADL*, *CPT1*, and *MCT2* transcripts were compared between neuroanatomical areas (in laser‐captured samples) or between serum‐exposure timepoints (in cell cultures) using a two‐tailed *t*‐test in Excel. Error bars are SEM.

### Serum‐Free Primary Culture of Mouse NSPCs

Adult NSPCs were isolated as described previously 18. Briefly, wild‐type C57BL/6 mice, 3 months of age, were transcardially perfused with ice‐cold saline. Brain tissue from the SVZ was mechanically and enzymatically dissociated with collagenase‐DNase solution. To remove debris, myelin and red blood cells, the cell suspension was mixed with a percoll solution and centrifuged. The isolated progenitor cells were grown in proliferation media, consisting of Dulbecco's modified Eagle's medium/F12 (Omega Scientific DM‐25, Tarzana, CA, www.omegascientific.com/) supplemented with 2 mM glutamine, 1% N2 (Life Technologies, Carlsbad, CA), 50 lg/ml heparin (Sigma, St Louis, MO, www.sigmaaldrich.com), 20 ng/ml epidermal growth factor (Peprotech, Rocky Hill, NJ, www.peprotech.com), and 20 ng/ml fibroblast growth factor‐2 (Peprotech, Rocky Hill, NJ). Cultures were passaged by mechanical dissociation in the presence of trypsin‐EDTA (Life Technologies, Carlsbad, CA) and used for in vitro experimentation between passages 3 and 12. Cultures contain a relatively homogenous, stable population of NSPCs (90% Nestin+; 80% CD133+) 18. In Supporting Information Figure 4, serum exposure involved the use of normal DM25 medium supplemented with 10% fetal bovine serum (FBS). In Figure 6, neuronal differentiation was achieved with 2% B27 supplement in Neurobasal Medium. All in vitro experiments were performed three times with separate biological replicates (each with three technical replicates), except Seahorse Analyzer experiments shown in Figure 3, which were performed three times with separate biological replicates (each with five technical replicates). Error bars are SEM.

### Extracellular Flux Analysis in Live Cells

For Figure 2, oxygen consumption rate (OCR) was measured using a flow culture system adapted for extracellular flux analysis as described 19. This system uses an ultrastable oxygen sensor based on the detection of the decay of the phosphorescent emission from an oxygen‐sensitive dye. For Figure 3, OCR was measured using the Seahorse XF24 Extracellular Flux Analyzer as described previously 20. OCR measurements were normalized to cell count per well and were averaged across three independent experiments each with multiple replicates. Results were statistically compared using two‐tailed *t*‐tests in Excel and plotted in Prism.

### Assessment of Cellular Proliferation and Viability

To quantify the fractions of actively cycling cells in the population, we used two methods: immunocytochemical labeling of KI67 and fluorescence‐activated cell sorting (FACS)‐based mitotic profiling. NSPCs were plated at a density of approximately 10,000 cells per well on coated glass coverslips for 24 hours in growth medium. Cells were then treated with 10 μl of dPBS, 5 mM etomoxir, or 5 mM linoleic acid, for a final concentration of 100 μM etomoxir and 100 μM linoleic acid. Immunohistochemistry was performed as above. TUNEL+ apoptotic cells were quantified as directed using a TdT Reagent Kit (Chemicon Temecula, CA, www.emdmillipore.com). All cells were costained with Hoechst diluted to 1 μg/ml in PBS containing 0.1% Triton X‐100. Coverslips were mounted onto glass slides using Citifluor antifade glycerol reagent. Fluorescence microscopy was performed using a Zeiss Axioskop with attached camera and Axiovision software. Treatment groups were compared using two‐tailed *t*‐tests in Excel.

To make additional measurements of cellular proliferation, we performed FACS‐based mitotic profiling. Cells were plated at a density of 10^6^ cells per plate and incubated at 37°C for 24 hours in growth medium. Cells were then treated with PBS, etomoxir at a final concentration of 70 μM or linoleic acid at a final concentration of 50 μM. Twenty‐four hours after treatment, cells were labeled with 2 μg/ml Hoechst in permeabilization solution for 1 hour, then subjected to fluorescence‐associated cell sorting using LSRII. Mitotic profile analysis was performed using ModFit software. Ten thousand readings were taken for each replicate. Treatment groups were compared using two‐tailed *t*‐tests in Excel.

To quantify the fraction of viable cells using an alternative method, we used the ViCell automated cell counter to measure total number of cells and the number of dead cells labeled positively with Trypan Blue dye. Cells were plated at 500,000 per plate and counted 48 hours later. Fractions of viable cells in each treatment group were compared using two‐tailed *t*‐tests in Excel.

### Pharmacological Inhibition of Fatty Acid and Lactate Transport In Vivo

Female C57B/6 mice between 5 and 7 weeks of age were injected intraperitoneally with 40 mg/kg etomoxir (Sigma, St Louis, MO) in PBS, 40 mg/kg alpha‐cyano‐4‐hydroxycinnamate (4‐CIN (Sigma, St Louis, MO)) in 1% methanol‐PBS, or with similar volumes of PBS. Animals were injected once per day for 3 days and killed on day 4, approximately 24 hours after the final injection. Perfusion and immunohistochemistry were performed as described above. Fluorescence microscopy was performed using a Zeiss Apoptome with attached camera and Axiovision software. Cell counts were compared using two‐tailed *t*‐tests in Excel.

### Lentiviral‐Mediated Gene Delivery of PGC1α and GFP In Vitro and In Vivo

To restore the relevant enzymatic machinery for fatty acid oxidation in aged NSPCs, we targeted NSPCs within SVZ for genetic modification by intracerebral injection of high‐titer lentivirus encoding either green fluorescent protein (GFP) alone or peroxisome proliferator‐activated receptor gamma coactivator 1 alpha (PGC1α) tagged with GFP. Two groups of mice, aged 21 months, were injected in SVZ with either a control virus expressing GFP or a virus expressing PGC1α tagged with GFP (*n* = 4 per group). Additional animals injected in a different location were used as controls to assess the extent of lentiviral infection and target gene expression. Animals were caged socially and killed by transcardial perfusion 2 months after surgery. Tissues were fixed, sectioned, stained, and visualized as described above. Cell counts were compared using two‐tailed *t*‐tests in Excel.

## Results

### Expression of Fatty Acid Oxidation Enzymes in Adult SVZ and Hippocampus

Brain energy metabolism has been shown to shift from beta‐oxidation of fatty acids to glucose‐based metabolism during weaning 15, 16. As a result, oxidation of fatty acids and ketone bodies in the central nervous system decreases drastically during maturation. We hypothesized that NSPCs however maintain a metabolic phenotype similar to less‐differentiated cells in the fetal brain. To test this question, we assessed whether enzymes required for fatty acid oxidation were present in NSPCs located within neurogenic areas of the adult brain.

**Figure 1:**
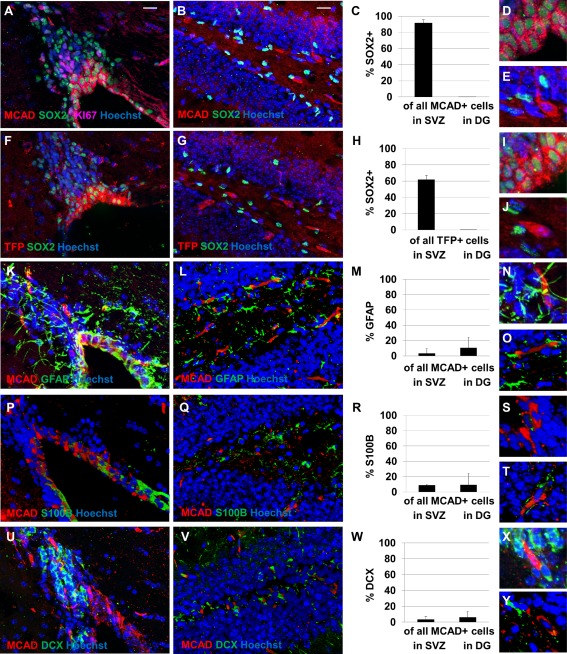
Neural stem/progenitor cells (NSPCs) in adult mouse subventricular zone (SVZ) express enzymes required for fatty acid oxidation. Cells expressing medium chain acyl CoA dehydrogenase (MCAD [**A–E]**) and trifunctional protein (TFP [**F–J]**) are observed in the SVZ (A, F) and hippocampal dentate gyrus (DG) (B, G). A majority of cells expressing these enzymes in SVZ are observed to be colabeled with SOX2, while none of the cells expressing fatty acid oxidation enzymes in DG are labeled with SOX2 (C, H). Higher‐magnification images of MCAD+ cells (D–E) and TFP+ cells (I–J) in SVZ (D, I) and DG (E, J) are shown. In SVZ, fewer than 5% of MCAD+ cells colabel with KI67 and fewer than 5% of KI67+ cells colabel with MCAD. Most MCAD+ cells (in red) do not colabel with glial fibrillary acidic protein (GFAP+) astrocytes **(K–O)**, S100B+ ependymal cells **(P–T)**, or doublecortin (DCX+) neuroblasts **(U–Y)**. All tissues were stained with the pan‐nuclear marker Hoechst (in blue) to demonstrate total cell number. Scale bar = 20 μm. Abbreviations: DCX, doublecortin; DG, dentate gyrus; GFAP, glial fibrillary acidic protein; MCAD, medium chain acyl CoA dehydrogenase; SVZ, subventricular zone; TFP, trifunctional protein.

Cells expressing medium chain acyl CoA dehydrogenase (MCAD) and trifunctional protein (TFP) are observed in the SVZ and hippocampal DG (Fig. 1). Positively labeled cells are also observed in the hypothalamic nuclei (not shown), as has been observed previously 21, 22. Cells in hippocampal DG that labeled positively for markers of fatty acid oxidation did not colabel with SOX2, a marker of progenitor cells. The cells expressing fatty acid oxidation enzymes in this area, therefore, do not appear to be a progenitor population. However, a majority of cells in SVZ containing these markers demonstrate colabeling with SOX2, suggesting that SOX2+ cells there are capable of fatty acid oxidation. However, very few MCAD+ cells in SVZ colabel with KI67, an S‐phase marker, suggesting that actively proliferating cells are not undergoing fatty acid oxidation. MCAD+ cells observed in SVZ also do not colabel with GFAP+ astrocytes, S100B ependymal cells, or doublecortin (DCX+) neuroblasts (Fig. 1). A negative control with no primary antibody is shown in Supporting Information Figure 1 and individual channel images are shown in Supporting Information Figures 2 and 3.

**Figure 2:**
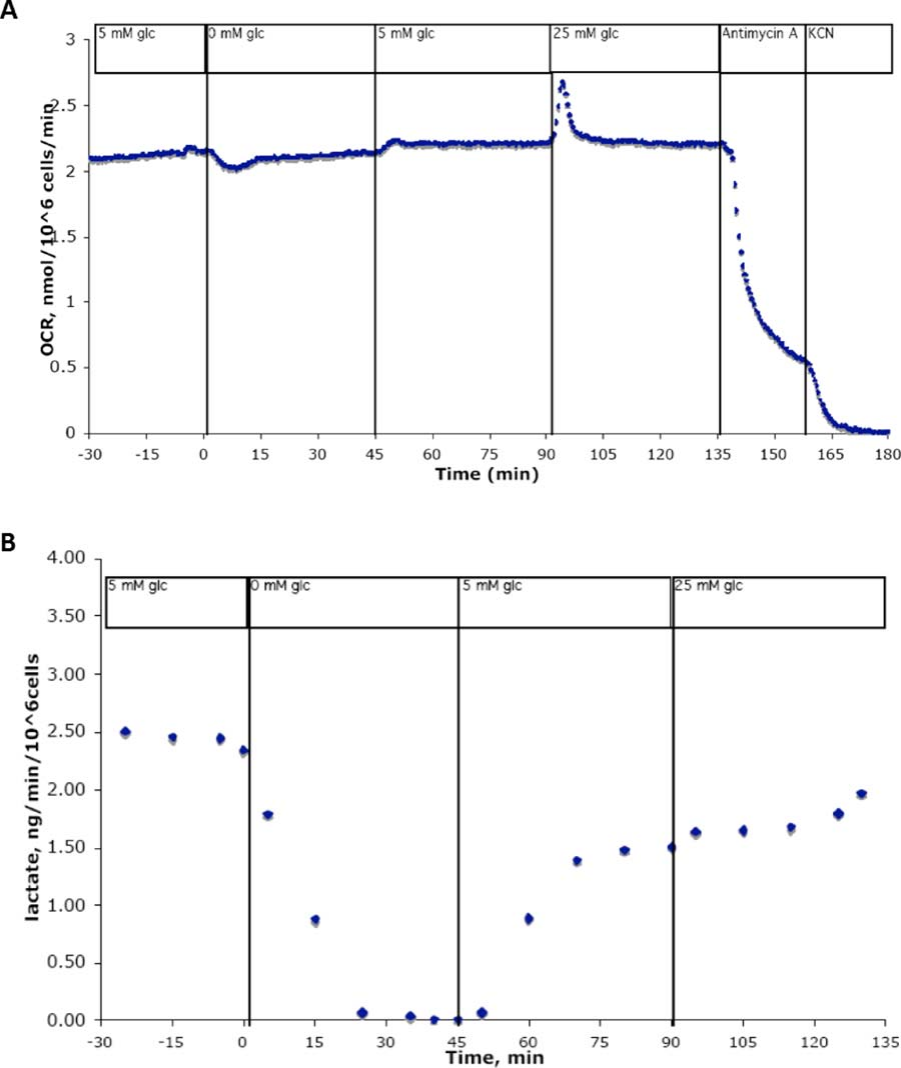
Neural stem cells do not require glucose to sustain aerobic respiration. Shown is a representative trace of cultured neural stem/progenitor cells (NSPCs) in a flow culture system outfitted for continuous metabolic analysis. Cells were exposed to differing concentrations of glucose over 210 minutes. Undifferentiated NSPCs do not sustain changes in oxygen consumption rate when glucose concentration is decreased to 0 mM, then returned to 5 mM, then increased to 25 mM for extended periods of time **(A)**, although simultaneous measures of lactate production drop to zero within 25 minutes of glucose removal **(B)**. Abbreviations: glc, glucose; KCN, potassium cyanide; OCR, oxygen consumption rate.

**Figure 3:**
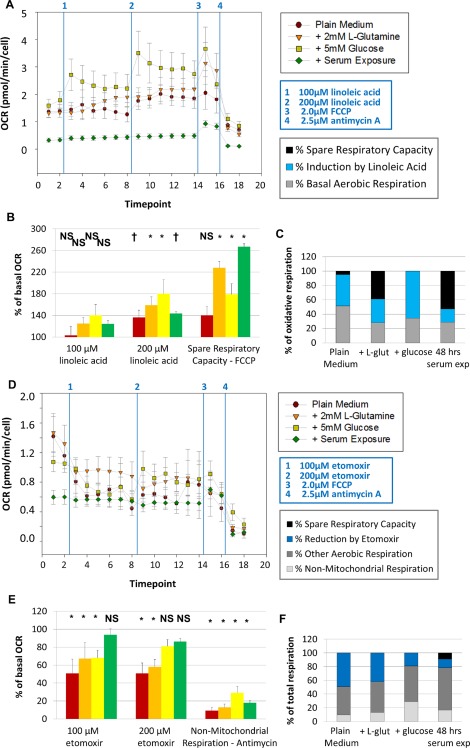
Fatty acid oxidation is a primary contributor to aerobic respiration in primary‐cultured neural stem/progenitor cells (NSPCs). The oxygen consumption rate (OCR [**A, E]**) and extracellular acidification rate (ECAR [**B, F**]) of primary‐cultured NSPCs were assessed using the seahorse analyzer. Baseline measurements were taken for cells in plain medium (− glutamine – glucose, red circles), for cells in medium + l‐glutamine (orange triangles), for cells in medium + glucose (yellow squares), and for cells exposed to 10% fetal bovine serum for 48 hours before the experiment (green diamonds). Cells were then treated with 100 μM linoleic acid, a polyunsaturated fatty acid. The concentration was then increased to 200 μM of linoleic acid. Cells were then treated with 2.0 μM FCCP, which induces maximal respiration, and 2.5 μM antimycin A, which completely inhibits aerobic respiration. (B): Cells do not show significant changes in OCR upon treatment with 100 μM linoleic acid, but cells do significantly increase OCR upon further addition of linoleic acid. **(C):** The fraction of maximal oxidative respiratory capacity that is used under basal conditions and after linoleic acid treatment is shown. **(D):** In a separate experiment, cells were treated with 100 μM etomoxir, an inhibitor of CPT1, the rate‐limiting enzyme in fatty acid oxidation; the concentration was then increased to 200 μM etomoxir. (E): All cells show significant decreases in OCR upon treatment with 100 μM etomoxir, except cells that had been exposed to serum. Cells in plain medium or in medium containing l‐glutamine maintain decreased OCR upon further addition of etomoxir, while cells with glucose show a partial recovery (E). (F): The fraction of total respiratory capacity attributed to nonmitochondrial respiration, fatty acid oxidation and other aerobic metabolism is shown. Measurements were taken every 9 minutes; data from three experiments were averaged together. †, *p* < 0.05; *, *p* < 0.01. Abbreviations: FCCP, carbonyl cyanide‐p‐trifluoromethoxyphenylhydrazone; OCR, oxygen consumption rate.

**Figure 4:**
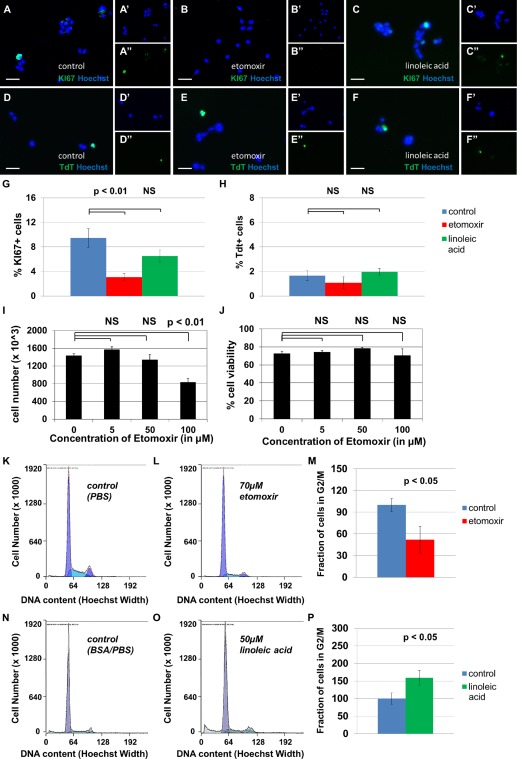
Inhibition of fatty acid oxidation decreases proliferation in cultured neural stem/progenitor cells (NSPCs), but does not affect cellular survival. Sample photomicrographs of cells treated with phosphate‐buffered saline (PBS) **(A, D)**, 100 μM etomoxir **(B, E)**, or 100 μM linoleic acid **(C, F)** are shown, stained with either KI67, an S‐phase cell cycle marker (A–C), or TdT, a marker of apoptosis (D–F). Separate channels are shown to display Hoechst, a pan‐nuclear marker (′), and TdT or KI67 (″). The fraction of KI67+ proliferating cells decreases after 24 hours treatment with etomoxir (*p* < 0.05, G) and does not change after 24 hours of treatment with linoleic acid (*p* > 0.05, not significant, G). The fraction of TdT+ apoptotic cells does not change significantly in either treatment group (*p* > 0.05, not significant, H). The total cell count after 48 hours is significantly decreased in the presence of etomoxir at 100 μM concentrations (*p* < 0.01, I) while cell viability remains unaffected (*p* > 0.05, not significant, J). Proliferation was also assessed by FACS‐based mitotic profiling of primary‐cultured NSPCs. In one experiment, cells were treated with vehicle control (PBS, K) or 70 μM etomoxir **(L)**. The fraction of cells in G2/M phase of the cell cycle decreased from control levels after 24 hours treatment with etomoxir (*p* < 0.05, M). In a separate experiment, cells were treated with vehicle control **(N)** or 50 μM linoleic acid **(O)**. The fraction of cells in G2/M phase of the cell cycle increased upon treatment with linoleic acid (*p* < 0.05, P). Scale bar = 20 μm.

Because MCAD is a critical enzyme in the beta‐oxidation of medium‐length fatty acids, all long‐chain and medium‐chain fatty acids will be processed by this enzyme. Therefore, if a cell undergoes fatty acid oxidation, it will contain this particular enzyme. Therefore, we have colabeled tissues with a mouse monoclonal antibody against MCAD in combination with polyclonal antibodies against GFAP, S100B, and DCX to investigate the identity of cells capable of fatty acid oxidation.

### Changes in Metabolic Gene Expression During Cellular Maturation

Differentiation into a mature neuron or astrocyte may involve modulating the metabolic machinery by upregulating gene products necessary for cell‐specific metabolic activity. Recent studies have shown that differentiation of embryonic day 14 (E14) neural stem cells into astrocytes, by addition of FBS to the culture medium, induces astrocyte‐specific metabolic characteristics such as glycogenolysis during the same timeframe necessary to develop typical morphological and immunohistochemical characteristics of astrocytes 23, 24. We hypothesized that differentiating cells in the adult brain may recapitulate developmental processes by becoming less dependent on beta‐oxidation of fatty acids and becoming more dependent upon carbohydrates and monocarboxylates.

To test whether metabolic machinery is remodeled in neurogenic areas of the adult brain, we performed laser‐capture microdissection and real‐time PCR to quantify metabolic transcripts in NSPCs from adult mouse SVZ, adult‐born neurons from the OB, and embryonically born neurons from cortex (Supporting Information Fig. 4A–4E). Cells taken from OB do not have significantly lower levels of transcripts for the fatty acid oxidation enzymes long‐chain acyl CoA dehydrogenase (*ACADL*) and Carnitine Palmitoyl Transferase I (*CPT1*), although cortical cells do have significantly lower levels of these transcripts compared with SVZ cells. Similar to cortical cells, cells taken from OB demonstrate significantly higher levels of transcripts for *MCT2* compared with SVZ cells.

Next, we aimed to establish a timeline of metabolic gene expression during in vitro differentiation of adult NSPCs over 24 hours of serum exposure (Supporting Information Fig. 4D–4L). We observed no changes in the quantity of transcripts for *ACADL* or *CPT1* during this time period. However, we did observe significant increases in *MCT2* transcripts. Together, these findings suggest that adult‐born neurons and glia acquire lactate transport machinery during differentiation but do not lose expression of enzymes required for fatty acid oxidation. This change in gene expression manifests early, as cells are only starting to show morphological and immunohistochemical signatures of differentiation.

### Substrates Used by NSPCs to Support Aerobic Respiration

Previous studies have shown that changes in glucose concentration cause strong, sustainable changes in the OCR of both neurons and astrocytes 12, 13. Here, we tested the response of NSPCs to changes in glucose concentration using a flow culture system to measure OCR and the extracellular acidification rate (ECAR) caused by continuous lactate production (an indirect measure of glycolytic activity). We observed that OCR remains steady through changes in glucose concentration, although lactate production drops to zero within 25 minutes of glucose removal (Fig. 2). Upon addition of glucose, a slight rise in OCR is observed, with the opposite effect observed upon removal of glucose. However, OCR returns to baseline levels shortly after any change in glucose concentration. These data suggest that neural stem cells do metabolize glucose, but are not dependent upon glucose to sustain aerobic respiration.

As primary‐cultured NSPCs do not require glucose to sustain aerobic respiration, they must have alternative metabolic fuel requirements than other cells within the adult brain. We hypothesized that fatty acids are instead used by NSPCs as substrates to power aerobic metabolism. To test this hypothesis, we compared the responses of undifferentiated and serum‐differentiated NSPCs to the polyunsaturated fatty acid linoleic acid and the drug etomoxir, which inhibits the rate‐limiting enzyme in fatty acid oxidation (CPT1). For these experiments, OCR was measured in live cells in real time using a Seahorse Analyzer. The addition of polyunsaturated fatty acids increases OCR significantly, even in the presence of alternative metabolic substrates such as l‐glutamine or glucose (Fig. 3A–3C). Pharmacological inhibition of fatty acid oxidation significantly decreases OCR in all groups (Fig. 3D–3F); however cells with access to glucose can recover OCR upon increased concentrations of etomoxir (Fig. 3D–3E). Cells that had been exposed to serum for 48 hours before experiments demonstrate an abrogated response to etomoxir, only slightly decreasing OCR in response to this inhibitor of fatty acid oxidation (Fig. 3D–3F). However, these cells are still capable of increasing respiratory rate in response to linoleic acid (Fig. 3A–3C).

### Effects of Pharmacological Inhibition of Fatty Acid Oxidation In Vitro

To test whether fatty acid oxidation plays a role in cellular survival and proliferation, we treated primary‐cultured mouse NSPCs with 100 μM etomoxir, 100 μM linoleic acid, or a vehicle control, then stained with either KI67, a cell cycle marker, or TdT, a marker of apoptosis (Fig. 4A–4H). The fraction of KI67+ proliferating cells decreases with etomoxir treatment, while the fraction of TdT+ apoptotic cells does not change significantly in either treatment group. In a separate assay, we found that doses of etomoxir approaching 100 μM decrease cell number significantly without affecting cell viability (Fig. 4I, 4J). We then subjected cells to FACS‐based mitotic profiling as an additional measure of proliferative activity (Fig. 4K–4P). We again observed significant decreases in the fraction of actively cycling cells upon etomoxir treatment. Since etomoxir decreases KI67+ index, the total cell count, and the fraction of cells in S+G2/M phase of the cell cycle, without affecting the TdT+ or Trypan Blue+ cell population, the function of this catabolic pathway appears to be important for the proliferation of neural stem cells. These data suggest that inhibition of fatty acid oxidation blocks proliferation of NSPCs without affecting rates of apoptosis.

### Effects of Pharmacological Inhibition of Fatty Acid Oxidation In Vivo

**Figure 5:**
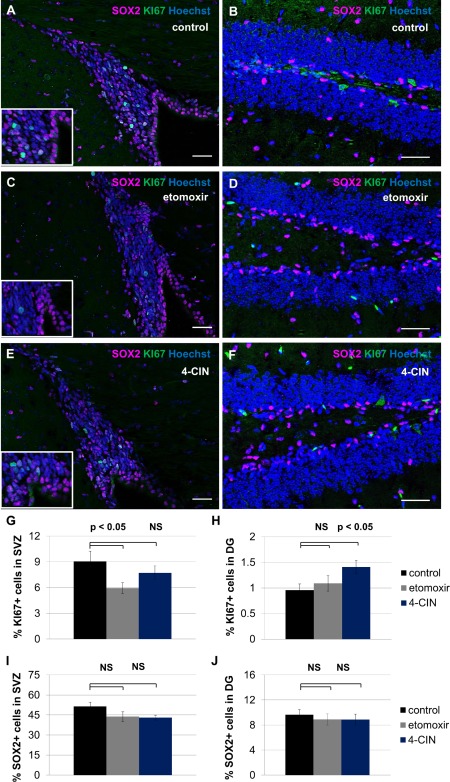
Inhibition of fatty acid oxidation decreases proliferation in subventricular zone (SVZ) but not hippocampal dentate gyrus. Adult mice received three injections of saline **(A, B)**, 40 mg/kg etomoxir **(C, D)** or 40 mg/kg alpha‐cyano‐4‐hydroxycinnamate (4‐CIN) **(E, F)**. The fraction of KI67+ cells and the fraction of SOX2+ cells was assessed within SVZ (A, C, E) and hippocampal dentate gyrus (DG) (B, D, F). Etomoxir treatment is associated with a significant decrease in KI67+ cells in SVZ (*p* < 0.05, G), while the effect of 4‐CIN on this population was not significant (*p* > 0.05, G). KI67+ cell number in the hippocampal DG was unchanged upon etomoxir treatment (*p* > 0.05, H), but significantly increased upon 4‐CIN treatment (*p* < 0.05, **I)**. The SOX2+ progenitor cell population was unaffected in both areas in both treatment groups (*p* > 0.05, not significant, J). Scale bar = 40 μm. Abbreviations: 4‐CIN, alpha‐cyano‐4‐hydroxycinnamate; DG, dentate gyrus; SVZ, subventricular zone.

To test whether inhibition of fatty acid oxidation blocks proliferation of NSPCs in vivo, female mice between 5 and 7 weeks of age were injected intraperitoneally on three consecutive days with 40 mg/kg etomoxir, 40 mg/kg 4‐CIN, or similar volumes of saline. The fraction of KI67+ cells and the fraction of SOX2+ cells were assessed immunohistochemically (Fig. 5). We observed a significant decline in KI67+ cells in the SVZ in etomoxir‐treated animals but no significant decline in KI67+ cells in this area in 4‐CIN‐treated animals. The SOX2+ population in SVZ is unaffected in both treatment groups. We observed no decline in KI67+ cells in the hippocampal dentate gyrus; indeed, 4‐CIN treatment was associated with a significant increase in mitotic index in this area. SOX2+ cells in DG are unaffected by treatment with either etomoxir or 4‐CIN. These data suggest that SVZ and hippocampal DG have different responses to metabolic inhibitors which might be predicted by enzyme expression patterns observed in Figure 1.

**Figure 6:**
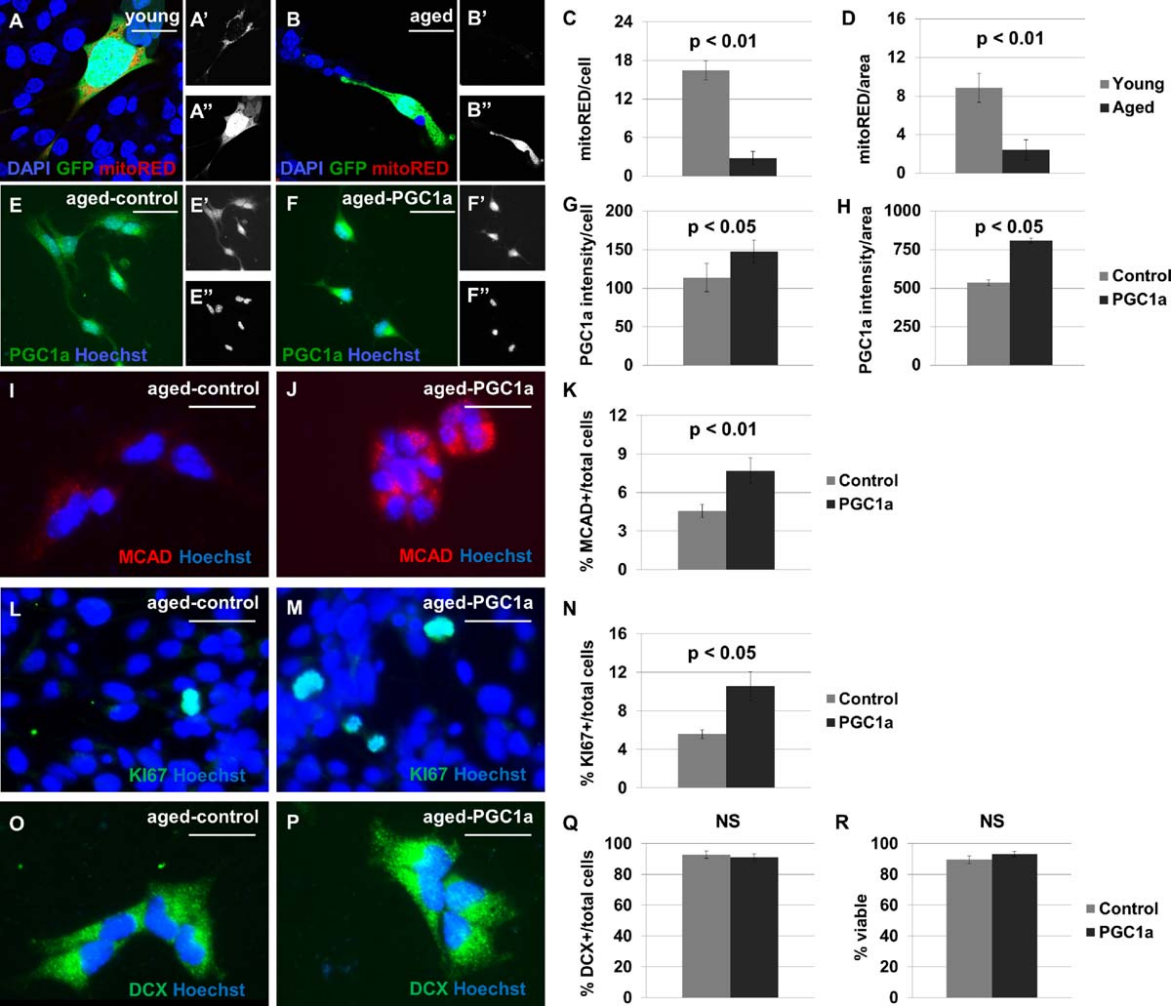
Ectopic proliferator‐activated receptor gamma coactivator 1 alpha (PGC1α) expression increases medium chain acyl CoA dehydrogenase (MCAD+) mitochondrial content and KI67+ mitotic index in cultured neural stem/progenitor cells (NSPCs). NSPCs from the young adult **(A)** and aged adult **(B)** mouse brain were labeled with MitoTracker Red; separate channels are shown to display this mitochondrial marker (′), and green fluorescent protein (GFP) (″). Fewer mitoRED+ mitochondria are observed in cultures from aged adult brain than from young adult brain (*p* < 0.01), when normalized per cell **(C)** or per unit area **(D)**. NSPC cultures derived from 18‐month‐old mouse brain were transduced with lentivirus to express GFP alone **(E)** or PGC1α‐GFP **(F)**. Separate channels are shown to display PGC1α expression (′), and the pan‐nuclear marker Hoechst (″). A 150% increase in nuclear PGC1α expression, compared with control, was observed by immunocytochemistry (*p* < 0.05, **G–H**). Cells expressing PGC1α demonstrate higher MCAD expression than control cells (*p* < 0.01, **I–K**). Cultures transduced with PGC1α‐GFP have a higher KI67+ index under growth conditions (*p* < 0.05, **L–N**). The cultures have no difference in doublecortin (DCX+) index (*p* > 0.05, **O–Q**) or trypan blue‐ viability (*p* > 0.05, **R**) after a 4‐day differentiation protocol. Scale bar = 20 μm. Abbreviations: DAPI, 4′,6‐diamidino‐2‐phenylindole; DCX, doublecortin; GFP, green fluorescent protein; MCAD, medium chain acyl CoA dehydrogenase; PGC1α, proliferator‐activated receptor gamma coactivator 1 alpha.

### Rescue of Neurogenic Activity in the Aged Brain by Increasing Aerobic Metabolic Capacity

Few neurons are produced in the aged brain, and the majority of neurogenic decline occurs by middle age. Aged NSPCs exhibit decreases in both mitochondrial content and aerobic respiratory activity 25. We hypothesized that expanding the aerobic capacity of the remaining population of NSPCs in the aged brain may boost neurogenesis and rescue regenerative activity. To do so, we performed lentiviral‐mediated rescue of a mitochondrial biogenesis factor within aged SVZ NSPCs. The target gene, *PGC1α*, induces mitochondrial biogenesis, coordinating an increase in mitochondrial number and metabolic gene expression, which has been shown to rescue oxidative respiration in cardiac myocytes 26, 27.

To test whether PGC1α affects metabolic gene expression in NSPCs, we overexpressed PGC1α‐GFP or GFP alone (as a control) in cultured NSPCs from the aged mouse brain. Lentiviral‐mediated transduction increases PGC1α expression and MCAD+ mitochondrial content (Fig. 6A–6K). Grown in proliferation medium, cultures transduced with PGC1α have a higher KI67+ index compared with controls (Fig. 6L–6N). Sustained in differentiation medium (2% B27 in Neurobasal Medium), cultures transduced with PGC1α have no difference in DCX+ index or viability (Fig. 6O–6R). These results indicate that PGC1α increases respiratory capacity and proliferative activity, but do not affect the propensity of cells to survive or undergo neuronal differentiation.

**Figure 7:**
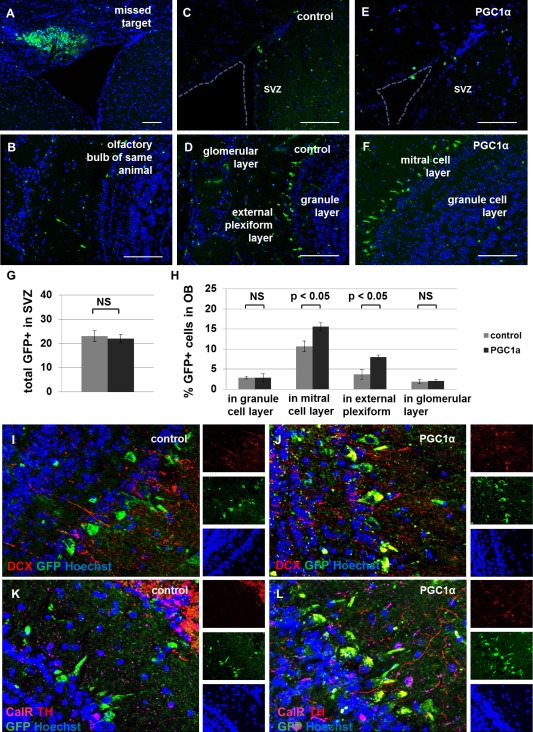
Ectopic proliferator‐activated receptor gamma coactivator 1 alpha (PGC1α) expression increases subventricular zone (SVZ)‐based neurogenesis in aged mice. Injection of lentivirus into a target location with nonmigratory cells demonstrates the extent of infection in the mouse brain 2 months post‐injection **(A)**. The olfactory bulb (OB) in this same animal is observed to contain few green fluorescent protein (GFP+) cells, as expected **(B)**. The remainder of mice were injected into the target location containing proliferative cells along the lateral ventricle. These animals have very few GFP+ cells in the SVZ when analyzed 2 months after inducing ectopic expression of GFP alone **(C)** or PGC1α + GFP **(E)**. Most GFP+ cells are observed in the OB 2 months after infection with virus encoding GFP alone **(D)** or PGC1α + GFP **(F)**. The total number of GFP+ cells in SVZ was not significantly different between groups (*p* > 0.05, **G**). The fraction of total cells expressing GFP was significantly higher in the PGC1α group compared with the control group in some areas of OB, specifically in the mitral cell layer and the external plexiform layer (*p* < 0.05, **H**) but not in the granule cell layer or the glomerular layer (*p* > 0.05, **H**). GFP+ cells in OB overexpressing PGC1α colabeled with doublecortin (DCX, **I–J**) and tyrosine hydroxylase (TH, **K–L**), but not calretinin (CalR, K–L). Scale bar = 100 μm. Abbreviations: DCX, doublecortin; GFP, green fluorescent protein; OB, olfactory bulb; PGC1α, proliferator‐activated receptor gamma coactivator 1 alpha; SVZ, subventricular zone; TH, tyrosine hydroxylase.

We targeted NSPCs within SVZ for genetic modification by intracerebral injection of high‐titer lentivirus tagged with GFP. Two groups of mice, aged 21 months, were injected with either the control virus expressing GFP or the virus expressing PGC1α tagged with GFP under the same constitutive promoter. The animals were killed 2 months after surgery. We observed similar numbers of GFP+ cells remaining in the SVZ of control and experimental animals; however, ectopic expression of PGC1α was associated with an increased number of GFP+ cells in some areas of the OB, specifically the mitral cell layer and the external plexiform layer (Fig. 7A–7H). While GFP+ cells in the OB of control animals did not colabel with neuronal markers, PGC1α‐overexpressing GFP+ cells colabeled with DCX and tyrosine hydroxylase (TH) (Fig. 7I–7L). These data suggest that increased metabolic capacity can promote the regenerative potential of aged NSPCs to a limited extent.

## Discussion

Molecular mechanisms shown to regulate the rate of adult neurogenesis include both cell‐intrinsic factors, such as tumor suppressor proteins 28, 29, 30, and factors within the microenvironment, such as growth factors 31. In addition, some behaviors affect neurogenic activity. For example, voluntary exercise has been shown to increase cellular proliferation in the adult hippocampus 2 while diets high in monounsaturated fatty acids decrease the BrdU+ proliferative cell index in the adult hippocampus 32. Although molecular pathways within the cell and factors within the microenvironment have been proposed to regulate the rate of adult neurogenesis 33, a blood‐borne factor directly linking behavioral activity with neurogenic activity has not been identified. Metabolic fuel availability may provide such a link. Manipulating the fuel availability of neural stem cells may indeed uncover mechanisms by which organismal behavior, energy consumption, and cellular activity are coupled in the adult mammalian brain.

In this study, we identify the metabolic fuel requirements of NSPCs and the underlying enzymatic machinery in these cells and we describe how both the enzymatic machinery and the physiological response to catabolic substrates changes during the process of differentiation. In addition, we tested whether the availability of metabolic substrates, particularly fatty acids, can limit proliferative activity in vivo using pharmacological and genetic methods.

Fatty acids are present in the bloodstream of adult mammals, commonly bound to serum albumin. The concentration of free fatty acids in human blood has been estimated at 444 μM, or 74% of albumin concentration estimated at 600 μM 34. The total concentration of fatty acids in human serum has been estimated at 3290 μg/ml, including saturated fats, monounsaturated fatty acids and polyunsaturated fatty acids 35. A full 30% of these fatty acids (1009 μg/ml) in human blood plasma are in the form of linoleic acid, which is present in quantities approaching 3.6 mM. Linoleic acid is, therefore, a readily available metabolic substrate.

Fatty acids can enter a cell directly or be produced within the cell itself. Indeed fatty acids have been shown to be produced endogenously in adult NSPCs 36. These fatty acids can be used as raw materials to build cell membranes or be metabolized into cannabinoids, eicosenoids, and other bioactive agents. Alternatively, they can be used to produce energy within the mitochondrion by a process called beta‐oxidation. To enter the mitochondrion, a fatty acid must be esterified to acyl CoA by acyl CoA synthetase. Within the intermembrane space, the fatty acyl‐CoA is then converted to an acyl‐carnitine by CPT1 and is transported across the inner mitochondrial membrane by carnitine translocase. Once inside the mitochondrial matrix, the carnitine is coverted back into a fatty acyl‐CoA. This molecule is first oxidized by a chain‐length‐specific acyl CoA dehydrogenase (i.e., MCAD, LCAD), in the process reducing FAD to FADH_2_. The resulting molecule is hydrated, oxidized again, and then cleaved into a fatty acyl‐CoA and acetyl‐CoA in a final step called ketothiolysis. These three steps are performed by the same enzyme, TFP. The acetyl‐CoA molecule is used in other bioenergetic pathways such as the Kreb's Cycle, while the remaining fatty acyl‐CoA (now two carbons shorter than the original molecule) restarts the four‐step process of beta‐oxidation. Critically, beta‐oxidation can be inhibited pharmacologically with etomoxir, which blocks the activity of the rate‐limiting transport enzyme CPT1.

The various experiments performed in this study yield a consistent image of the metabolic substrates preferred by NSPCs and their newly differentiated progeny. SOX2+ progenitor cells in SVZ but not hippocampal DG express enzymes required for fatty acid oxidation, and inhibition of this pathway in vivo decreases proliferative activity within SVZ but not DG. As expected, cellular differentiation, both in vivo and in vitro, leads to increased expression of the MCT. However, the same cells do not lose expression of fatty acid oxidation enzymes. Correspondingly, serum‐exposed NSPCs retain responsiveness to linoleic acid but are unaffected by inhibition of fatty acid oxidation. The differentiated cells therefore seem able to metabolize either glucose or fatty acids, depending on substrate availability, better than the undifferentiated cells. Undifferentiated NSPCs rely greatly upon fatty acid oxidation for their proliferative activity, since etomoxir treatment leads to significant decreases in aerobic respiratory activity, KI67+ index, and FACS mitotic profile. We observed significant increases after linoleic acid treatment in FACS‐based assays but not in KI67+ labeling index. This discrepancy may be due to the difference in a suspension assay versus a monolayer assay. A detergent effect of the fatty acid could have caused mitotic cells loosely adherent to the coverslip to be washed away during staining; since all cells were spun down and collected for FACS‐based mitotic profiling, such an occurrence would not have led to a loss of cells. The necessity of fatty acid oxidation for neurogenic activity was borne out by further experiments in vivo. The relative importance of fatty acids over lactate was demonstrated in a pharmacological study using etomoxir and 4‐CIN, respectively. The results in the SVZ were consistent with other findings in this study, supporting the requirement of fatty acid oxidation for proliferative activity. However, the results in the hippocampus were intriguing. It appears that inhibiting lactate metabolism with 4‐CIN actually boosts stem cell identity and activity in DG. Perhaps new neurons are forced to remain in cell cycle when rendered unable to use the preferred substrate of neurons. Further experiments are needed to understand the metabolic fuel requirements of NSPCs in DG.

The remaining population of stem‐like cells in the aged brain demonstrate decreased aerobic capacity. Specifically, aged NSPCs have decreased mitochondrial mass and lower baseline OCRs compared with young NSPCs 25. In this report, we show that expanding the aerobic capacity of the remaining population of NSPCs in the aged brain, by ectopically expressing PGC1α within the SVZ, increases mitotic activity in vitro and boosts new neuron number in some areas of the OB. PGC1α is a broad regulator of aerobic metabolism, increasing mitochondrial content, mitochondrial DNA replication, and metabolic gene expression 26, 27, 37. However, this factor can have other effects on a cell, particularly on neuronal gene expression. Interestingly, one recent study demonstrated reduced parvalbumin (PV) expression and a decreased number of PV interneurons in animals lacking PGC1α 38 while another study demonstrated reduced TH expression upon ectopic PGC1α expression in dopaminergic neurons 39. These studies suggest that PGC1α may play a critical role in establishing cellular identity in the central nervous system. The mechanisms by which catabolic strategies and mitochondrial remodeling link to neuronal physiological function remains an active area of study.

As catabolic activity is a fundamental characteristic of a cell, exquisitely optimized to meet energetic needs and constraints, the identification of metabolic substrates required by the adult neural stem cell is imperative to understanding the process of regeneration. It is possible that different cell types rely upon different bioenergetic fuels. Different neuronal and glial subtypes in the brain may have distinctive “batteries” which confer cell‐specific properties, like glycolytic fast‐twitch and fat‐burning slow‐twitch fibers in skeletal muscle tissue. In addition, molecular sensors that coordinate catabolic and anabolic activity within brain cells may contribute to sensing the local availability of bioenergetic substrates and adjusting physiological activity accordingly. Characterizing cell‐specific metabolic machinery may therefore inform on cellular identity and physiological function.

In light of this study, an improved understanding of metabolic limitations on cellular proliferation may aid strategies to boost regenerative potential in the injured or aged central nervous system. However, further testing is needed before an expansion of metabolic capacity can be considered as an approach to counter age‐related cognitive decline. In particular, it is important to assess whether such interventions can enhance cognition in animal models and whether any side effects are observed from augmenting proliferation in the endogenous neural stem/progenitor population.

## Conclusions

Endogenous neural stem cells are excellent therapeutic targets, retaining the capacity to rebuild and repair tissue in the adult brain by producing new neurons and astrocytes. A great aim of regenerative medicine is to identify methods to support the self‐renewal of this population in the aged or diseased brain. Regulation of metabolic fuel availability could prove a powerful tool in promoting or constraining regeneration in the central nervous system.

Cell division is an energetically expensive task. While mature cells in the adult brain have been shown to rely upon carbohydrates such as glucose, the bio‐energetic substrates utilised by neural stem cells have heretofore been uncharacterized. In this study, we show that neural stem cells in the adult brain rely largely on fatty acids to fuel energy production. Using a combination of techniques, we demonstrate that fatty acid oxidation is required for neural stem cells to sustain aerobic respiration and proliferative activity. Our results have important implications for the study of stem cell biology, by introducing the role of bio‐energetic capacity as a limiting factor in neurogenic activity and as a defining factor in cellular identity.

## Author Contributions

E.A.S.: designed, performed, and analyzed experiments, wrote the manuscript; R.M.: performed cell culture, tissue cryosectioning, immunohistochemistry, and microscopy for experiments shown in Figures 4 and 5, contributed in editing the manuscript; I.R.S.: contributed intellectually and designed experiments shown in Figures 2 and 3, contributed in editing the manuscript; A.T.: contributed intellectually and performed surgeries for experiments shown in Figure 7, contributed in editing the manuscript; S.M.: assisted with Seahorse experiments shown in Figure 3, contributed in editing the manuscript; P.J.H.: initial experiments (metabolic physiology shown in Fig. 2 and ViCell/FACS analysis shown in Fig. 4) were performed in his laboratory, contributed in editing the manuscript; D.M.T.: all other experiments were performed in his laboratory, provided funding, resources, and intellectual support, contributed in editing the manuscript.

## Disclosure of Potential Conflicts of Interest

The authors indicate no potential conflicts of interest.

## Supporting information

Supplementary Information Figure 1

Supplementary Information Figure 2

Supplementary Information Figure 3

Supplementary Information Figure 4

Supplementary Information Table 1

Supplementary Information Table 2 Page 1

Supplementary Information Table 2 Page 2

Supplementary Information Table 2 Page 3

Supplementary Information

Supplementary Information
