# Supplementary material for: Neural Stem Cells in the Adult Subventricular Zone Oxidize Fatty Acids to Produce Energy and Support Neurogenic Activity
Source: Stem Cells. 2015 Jun 4;33(7):2306–19. doi: 10.1002/stem.2042 (PMC4478223; doi:10.1002/stem.2042)
Supplement: Supplementary file 9 — Supplementary Information [file STEM-33-2306-s009.docx]

**Supplemental Materials And Methods**

***Immunohistochemistry***

All experiments were performed as approved by the University of Washington Institutional Animal Care and Use Committee (IACUC) and United Kingdom Home Office Project Licence Number 60/4386. Female C57BL/6 mice were housed at 21°C with access to food and water ad libitum. For tissue collection, wild-type C57BL/6 mice, 3 months of age, were overdosed with Beuthanasia and transcardially perfused with ice-cold saline followed by 4% paraformaldehyde in PBS. Brains were post-fixed overnight, moved to 10% sterile sucrose solution for several hours, then stored in 30% sterile sucrose solution until ready for cryosectioning. Tissue slices were subjected to 0.01M sodium citrate at 100°C for ten minutes for antigen retrieval. Sections were then rinsed with PBS containing 0.1% Triton X-100 (PBST). Non-specific staining was blocked for 2 hours in PBS with 0.1% Triton X-100 and 5% donkey serum (blocking solution). Sections were incubated overnight at 4°C with appropriate antibodies diluted in blocking solution (details in Supplemental Table 1A). Sections were then rinsed with PBST. The appropriate secondary antibodies were diluted by 1:250 in blocking solution and placed on sections for 2 hours at room temperature (details in Supplemental Table 1B). Sections were rinsed and co-stained with Hoechst diluted to 1μg/mL in PBST. Coverslips were mounted over sections using Citifluor anti-fade glycerol reagent. Fluorescence microscopy was performed using a Zeiss Apoptome Microscope with attached camera and Axiovision software. Cells were counted from photomicrographs of 10μm-thick sections (approximately every eighth section across the entirety of the relevant brain region). Fixed brain tissue from four mice was used for this characterisation study. Error bars are SD.

***Laser-Capture Microdissection***

Cells within subventricular zone (SVZ), olfactory bulb (OB), or cortex were microdissected using the Zeiss PALM MicroBeam Laser-Capture Microdissection Microscope. Cells were collected in 12μl of RNAqueous solution. After catapulting and collection, tubes were centrifuged. mRNA was isolated using the RNAqueous MicroKit (Ambion AM1931). Transcripts were reverse-transcribed to cDNA using SuperScript III (Invitrogen, 18080-051). The reaction conditions were as follows: 25°C for 10 minutes, 50°C for 50 minutes, 85°C for 5 minutes. The reaction was then chilled on ice. RNAse H was added to each tube and allowed to incubate for 20 minutes. The samples were then stored at -20°C until ready for analysis with real-time PCR.

***Real-Time PCR***

Real-time PCR was used to quantify metabolic transcripts in laser-captured tissue samples and serum-exposed dissociated cells. The real-time PCR was based on the procedure described previously [^17^](#_ENREF_17) which quantifies fluorescence emitted by dyes VIC and FAM which are conjugated to probes complementary to cDNA reverse-transcribed from mRNA transcripts. A 20μl reaction volume contained 10μl of TaqMan Universal Master Mix II with UNG (Applied Biosystems), 4μl of DEPC dH_2_O and 1μl each of the following: forward primer, reverse primer, and VIC- or FAM- probe corresponding to one of the following regions: ACADL, CPT1, and MCT2. (Life Technologies). 1μl samples of cDNA (laser-microdissected tissue or dissociated cells) were run in triplicate, alongside 5 point 10-fold dilutions of a liver homogenate sample and a blank (dH_2_O). The assay was performed on the StepOnePlus™ Real-Time PCR System (Applied Biosystems). The PCR reaction conditions used were: incubation at 50°C for 2 minutes followed by denaturation at 95°C for 10 minutes, after which amplification occurred over 40 cycles consisting of 15 seconds at 95°C and 1 minute at 60°C. Samples from each neuroanatomical area or serum-exposure timepoint were each run in triplicate to quantify total copy number. Each sample was normalized to the standard curve, using the equation VALUE = (CT – INTERCEPT)/SLOPE. These values were normalized to B-actin. Total copy number of cDNA representing ACADL, CPT1, and MCT2 transcripts were compared between neuroanatomical areas (in laser-captured samples) or between serum-exposure timepoints (in cell cultures) using a two-tailed t-test in Excel. Error bars are SEM.

***Serum-free primary culture of mouse neural stem/progenitor cells***

Adult NSPCs were isolated as previously described ^[18](#_ENREF_18)^. Briefly, wild-type C57BL/6 mice, 3 months of age, were transcardially perfused with ice-cold saline. Brain tissue from the subventricular zone was mechanically and enzymatically dissociated with collagenase-DNase solution. To remove debris, myelin and red blood cells, the cell suspension was mixed with a percoll solution and centrifuged. The isolated progenitor cells were grown in proliferation media, consisting of Dulbecco’s modified Eagle’s medium/F12 (Omega Scientific DM-25, Tarzana, CA) supplemented with 2 mM glutamine, 1% N2 (Gibco, Carlsbad, CA), 50 lg/ml heparin (Sigma, St Louis, MO), 20 ng/ml epidermal growth factor (Peprotech, Rocky Hill, NJ), and 20 ng/ml fibroblast growth factor-2 (Peprotech, Rocky Hill, NJ). Cultures were passaged by mechanical dissociation in the presence of trypsin-EDTA, and used for in vitro experimentation between passages 3 and 12. Cultures contain a relatively homogenous, stable population of NSPCs (90% Nestin+; 80% CD133+) ^[18](#_ENREF_18)^. In Supp Figure 4, serum exposure involved the use of normal DM25 medium supplemented with 10% fetal bovine serum. In Figure 6, neuronal differentiation was achieved with 2% B27 supplement in Neurobasal Medium. All *in vitro* experiments were performed three times with separate biological replicates (each with three technical replicates), except Seahorse Analyzer experiments, which were performed three times with separate biological replicates (each with five technical replicates) and intensity measurements shown in Figure 6, which were performed three times with separate biological replicates (each with six technical replicates). Error bars are SEM. Treatment groups were randomly assigned to wells and quantitative analysis of fixed coverslips was performed by a blinded researcher. Staining procedures were performed on entire cohorts at the same time; photomicrographs were taken under identical exposure conditions by a blinded researcher, and identical thresholds were used to quantify labelled cells throughout a given experiment (again, by a blinded researcher).

***Extracellular flux analysis in live cells***

For Figure 2, oxygen consumption rate (OCR) was measured using a flow culture system adapted for extracellular flux analysis as described [^19^](#_ENREF_19). This system utilizes an ultrastable oxygen sensor based on the detection of the decay of the phosphorescent emission from an oxygen-sensitive dye. This system allows for sensitive measurements of inflow and outflow oxygen tension, with control over both aqueous and gaseous composition of the inflow. In addition, outflow from the metabolic chamber can be collected and subjected to analysis for additional metabolites (i.e. lactate).

For Figure 3, OCR was measured using the Seahorse XF24 Extracellular Flux Analyzer as previously described ^[20](#_ENREF_20)^. Mouse neural stem/progenitor cells were plated in XF24 cell culture plates (Seahorse Bioscience) at 10^5 cells/well and incubated for 48 hours at 37°C with 5% CO_2_. One row of cells contained 10% fetal bovine serum (FBS). Four blank wells were also included in the plate. On the day of experimentation, each well was replaced with bicarbonate-free low-buffered medium (Sigma, D5030); this medium contained one of the following: no supplement, 5mM glucose, 2mM L-glutamine, or 1% FBS in the wells which had been exposed to 10% FBS. Cells were incubated for one hour at 37°C with atmospheric CO_2_. 10X concentrations of substrates were prepared and placed in the appropriate port to be injected automatically at times indicated. Substrates were prepared from stock concentrations as follows: 1mM linoleic acid in HBSS for a final concentration of 100μM (this reagent was added a second time to achieve a concentration of 200μM), 1mM etomoxir in HBSS for a final concentration of 100μM (this reagent was added a second time to achieve a concentration of 200μM), 20μM FCCP in Seahorse Medium for a final concentration of 2.0 μM and 25μM Antimycin-A in Seahorse Medium for a final concentration of 2.5μM. Basal OCR was established before the first treatment. Each timepoint included five minutes of rest, one minute of mixing, and three minutes of measuring. OCR measurements were later normalized to cell counts. All timepoints for each treatment (baseline = 1-3; 100μM linoleic acid or etomoxir = 4-9; 200μM linoleic acid or etomoxir = 10-15; FCCP = 16-17; Antimycin A = 18-19) were averaged to achieve stable readings for further analysis. The normalized OCR measurements were averaged across three independent experiments each with multiple replicates. Results were statistically compared using two-tailed t-tests in Excel and plotted in Prism. Error bars are SEM.

***Assessment of cellular proliferation and viability***

To quantify the fractions of actively cycling cells in the population, we employed two methods: immunocytochemical labelling of KI67 and FACS-based mitotic profiling. For KI67 labelling, 13 mm glass coverslips were placed in 24 well plates and coated with 10μg/mL laminin in dPBS for two hours at 37°C. Mouse neural stem/progenitor cells were plated at a density of ~10,000 cells per well on coated glass coverslips for 24 hours in growth medium. Cells were then treated with 10μL of dPBS, 5mM etomoxir or 5mM linoleic acid, for a final concentration of 100μM etomoxir and 100μM linoleic acid. Etomoxir was diluted in PBS. Linoleic acid was prepared as follows: 15mg of linoleic acid was diluted in 5mL dH2O and heated at 55°C until dissolved; 546mg of bovine serum albumin was diluted in 5mL dPBS and vortexed until dissolved; the two reagents were mixed, sterile-filtered, and stored at -20°C. The final dilution of BSA in cell culture medium was 0.1%. 24 hours after treatment, cells were fixed in 4% paraformaldehyde at room temperature for 5 minutes and rinsed twice with phosphate-buffered saline (PBS). Coverslips with attached cells were then placed in humidified chambers (large tissue culture plates) for staining procedures. Immunohistochemistry was performed as above. TUNEL+ apoptotic cells were quantified as directed using a TdT Reagent Kit (Chemicon S7160). All cells were co-stained with Hoechst diluted to 1μg/mL in PBS containing 0.1% Triton X-100. Coverslips were mounted onto glass slides using Citifluor anti-fade glycerol reagent. Fluorescence microscopy was performed using a Zeiss Axioskop with attached camera and Axiovision software. Samples were photomicrographed and quantified in a blinded fashion and compared using two-tailed t-tests in Excel.

To make additional measurements of cellular proliferation, we performed FACS-based mitotic profiling. Cells were plated at a density of 10^6 cells per plate and incubated at 37°C for 24 hours in growth medium. Cells were then treated with dPBS, etomoxir at a final concentration of 70μM or linoleic acid at a final concentration of 50μM. 24 hours after treatment, cells were labelled with 2μg/mL Hoechst in the following permeabilization solution: 0.1M Tris (pH 7.4), 0.154M NaCl, 0.5mM MgCl2, 1mM CaCl2, 0.1% Triton X-100, 0.2% BSA. Cells were incubated in this solution for one hour, then subjected to fluorescence-associated cell sorting using LSRII. Mitotic profile analysis was performed using ModFit software. 10,000 readings were taken for each replicate. Treatment groups were compared using two-tailed t-tests in Excel.

To quantify the fraction of viable cells using an alternative method, we employed the ViCell automated cell counter to measure total number of cells and the number of dead cells labelled positively with Trypan Blue dye. Cells were plated at 500,000 per plate and counted 48 hours later. Fractions of viable cells in each treatment group were compared using two-tailed t-tests in Excel.

***Pharmacological inhibition of fatty acid and lactate transport* in vivo**

Female C57B/6 mice between 5-7 weeks of age were injected intraperitoneally with 40mg/kg etomoxir in PBS, 40mg/kg alpha-cyano-4-hydroxycinnamate (4-CIN) in 1% methanol-PBS, or with similar volumes of PBS. Animals (n = 6 per group) were injected once per day for three days and sacrificed on the fourth day, approximately 24 hours after the final injection. Perfusion and immunohistochemistry were performed as described above. Fluorescence microscopy was performed using a Zeiss Apoptome with attached camera and Axiovision software. Treatment groups were randomly assigned to animals and quantitative analysis of fixed tissues was performed by a blinded researcher. Specifically, staining procedures were performed on entire cohorts at the same time, photomicrographs were taken under identical exposure conditions by a blinded researcher, and identical thresholds were used to quantify labelled cells throughout a given experiment by a blinded researcher. Cells were counted from photomicrographs of 10μm-thick sections (approximately every eighth section across the entirety of the relevant brain region). Cell counts were compared using two-tailed t-tests in Excel.

***Lentiviral-mediated gene delivery of PGC1a and GFP* in vitro and in vivo**

To restore the relevant enzymatic machinery for fatty acid oxidation in aged NSPCs, we targeted neural stem/progenitor cells within SVZ for genetic modification by intracerebral injection of high-titer lentivirus encoding either GFP alone or peroxisome proliferator-activated receptor gamma co-activator 1 alpha (PGC1α) tagged with GFP. The PGC1α gene, whose product coordinates mitochondrial biogenesis and metabolic gene expression, was obtained from a carrier plasmid provided by Professor Bruce Spiegelman (Addgene, Plasmid 1026). This gene was subcloned into a lentivector plasmid (SBI CD526A-1) under a ubiquitous promoter (EF1) and tagged with green fluorescent protein (GFP). The gene of interest and GFP were separated by a T2A sequence which allows the two genes to be transcribed together under control of a single promoter but translated separately via a ribosomal skip mechanism. The parental plasmid was used as a GFP-expressing control. Plasmids, containing an antibiotic resistance gene, were amplified in E. coli and purified using a MaxiPrep kit (Qiagen 12362). Each plasmid was transfected along with three housekeeping genes (pMDG, pRSV-REV and pMDLg/pRRE) into HEK293T cells by calcium chloride co-precipitation. HEK293T cells assemble the third-generation lentiviral vector and release it into the media. The media was then concentrated by ultracentrifugation.

MitoTracker Red, tagged with GFP, was transduced into cell cultures to quantify mitochondrial content. After lentiviral-mediated transduction, PGC1α and MCAD expression were assessed by immunocytochemistry, as described above. Cultured cells were grown on coverslips, then fixed and stained as previously described; photomicrographs were taken by a blinded researcher and these raw images were quantified using ImageJ. Staining procedures were performed on entire cohorts at the same time; photomicrographs were taken under identical exposure conditions; identical thresholds were used to complete quantitation of labelled cells in a given experiment.

For in vivo experimentation, two groups of mice, aged 21 months, were injected with either a control virus expressing GFP or a virus expressing PGC1α tagged with GFP (n = 4 per group). The stereotactic coordinates were as follows: AP 1.1, ML 0.8, DV -2.1 and -1.7. Injecting into the SVZ ensures viral uptake by stem and progenitor cells. Additional animals injected in a different location were used as controls to assess the extent of lentiviral infection and target gene expression. Animals were caged socially and euthanized by transcardial perfusion two months after surgery. Tissues were fixed, stored, and cryosectioned as described above. Fluorescence microscopy was performed using a Zeiss Apoptome with attached camera and Axiovision software. Treatment groups were randomly assigned to animals and quantitative analysis of fixed tissues was performed by a blinded researcher as described in the previous section. Cell counts were compared using two-tailed t-tests in Excel.
