# Supplementary material for: Neural Stem Cells in the Adult Subventricular Zone Oxidize Fatty Acids to Produce Energy and Support Neurogenic Activity
Source: Stem Cells. 2015 Jun 4;33(7):2306–19. doi: 10.1002/stem.2042 (PMC4478223; doi:10.1002/stem.2042)
Supplement: Supplementary file 10 — Supplementary Information [file STEM-33-2306-s010.docx]

**Supplemental Figures**

**Supplemental Figure 1. Negative control for immunohistochemistry (no primary antibody).**

**Supplemental Figure 2. Individual channel images demonstrating co-labeling of fatty acid oxidation enzymes with SOX2 in subventricular zone (SVZ) and hippocampal dentate gyrus (DG).**

**Supplemental Figure 3. Individual channel images demonstrating a lack of MCAD co-labeling with alternative cell markers in SVZ.**

**Supplemental Figure 4. NSPCs acquire lactate transport machinery but do not lose fatty acid oxidation machinery during differentiation.**

**Supplemental Table 1. Antibodies used for immunohistochemical assays in this study.**

**Supplemental Table 2. Means, standard errors, and p-values for all experiments in this study.**

**
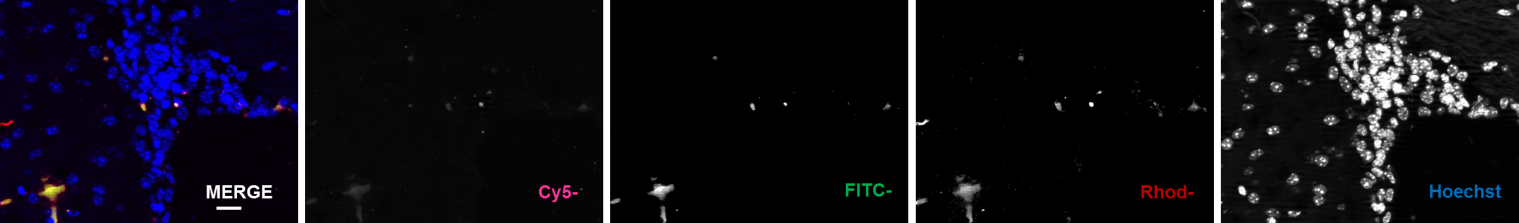
**

**Supplemental Figure 1. Negative control for immunohistochemistry (no primary antibody).** Scale bars represent 20 μm.

**
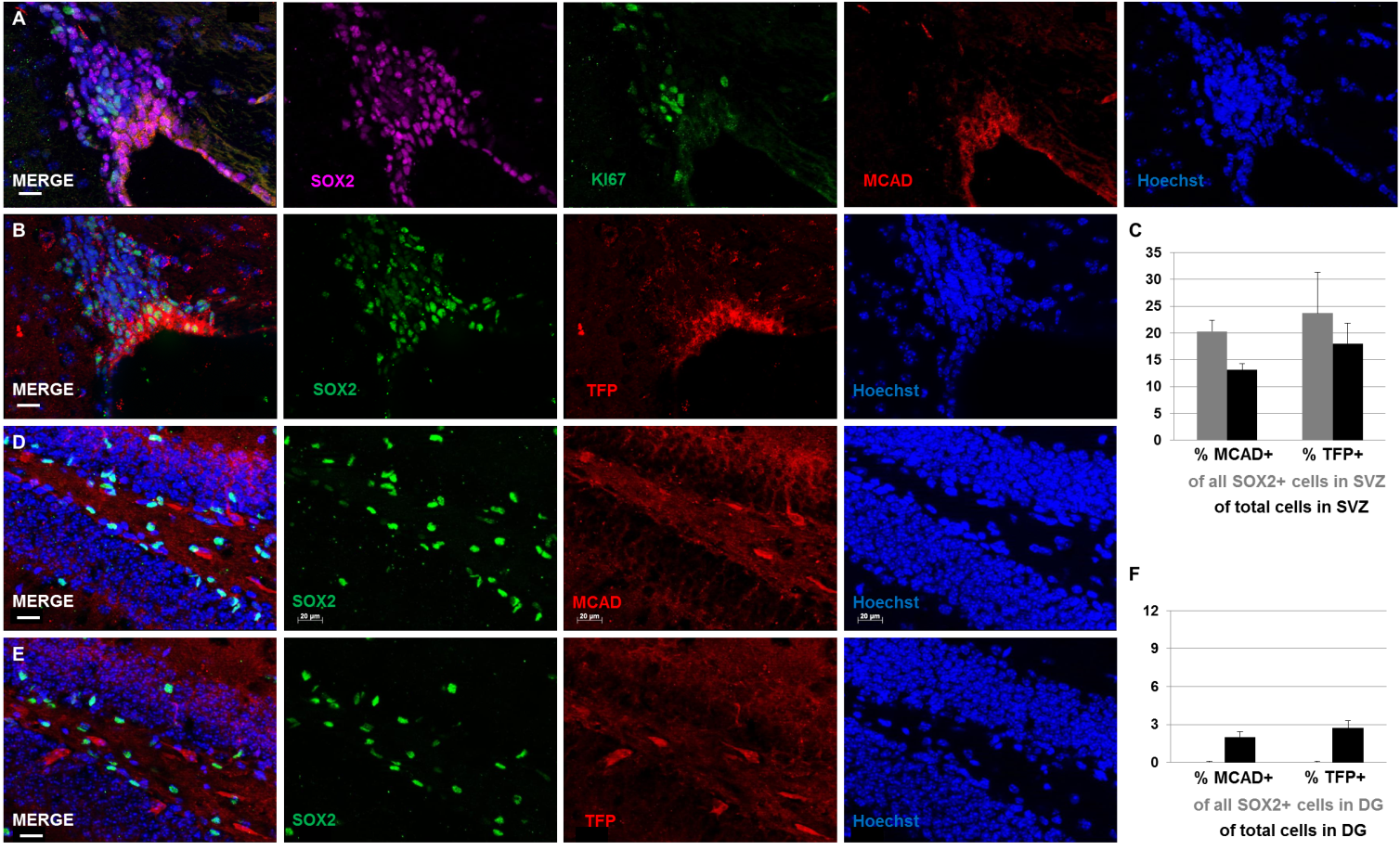
**

**Supplemental Figure 2. Individual channel images demonstrating co-labeling of fatty acid oxidation enzymes with SOX2 in subventricular zone (SVZ) and hippocampal dentate gyrus (DG).** Cells expressing Medium Chain Acyl CoA Dehydrogenase (MCAD, A and D) and TriFunctional Protein (TFP, B and E) are observed in the subventricular zone (A-C) and hippocampal dentate gyrus (D-F). Individual channels are shown for merged images presented in Figure 1. A fraction of SOX2+ cells co-label with each of these markers in SVZ (C) but not DG (F). Y-axis shows the % of fatty acid oxidation enzyme expression of all SOX2+ cells (in gray) and of total cells (in black). Scale bars represent 20 μm.

**
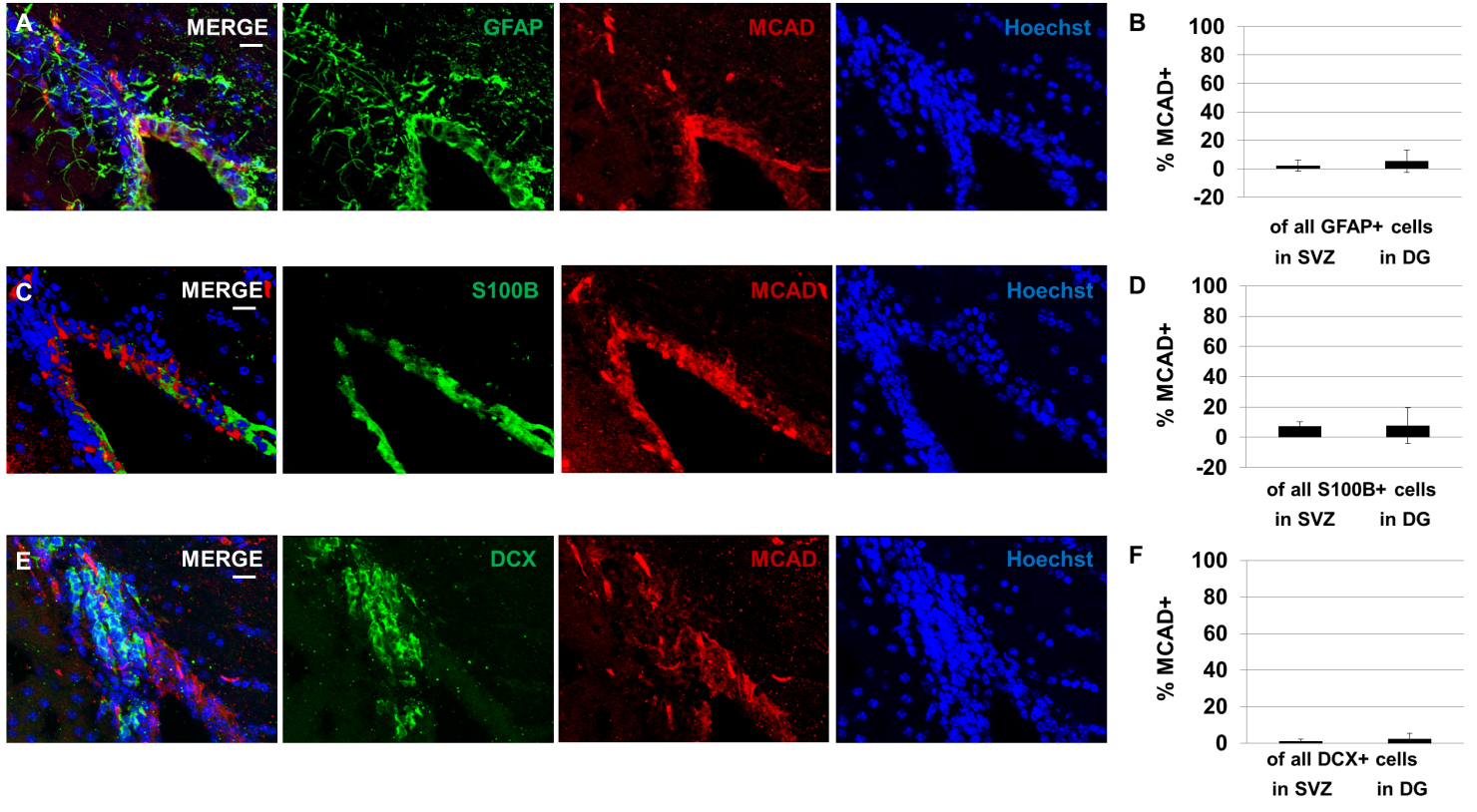
**

**Supplemental Figure 3. Individual channel images demonstrating a lack of MCAD co-labeling with alternative cell markers in SVZ.** Cells expressing Medium Chain Acyl CoA Dehydrogenase (MCAD) in the subventricular zone do not co-label with GFAP (A,B), S100B (C, D), or DCX (E, F). Individual channels are shown for merged images presented in Figure 1. Scale bars represent 20 μm.

**
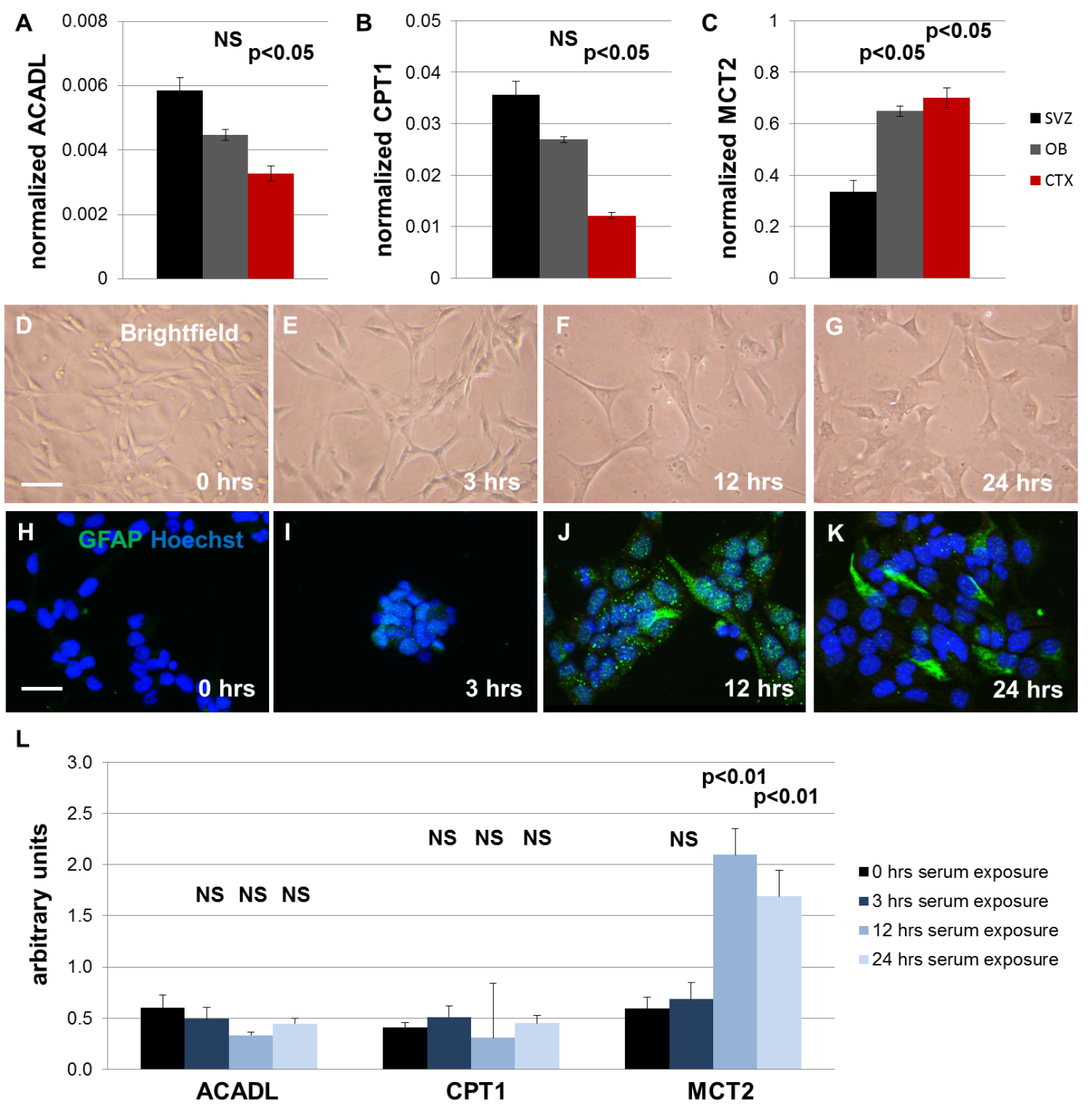
**

**Supplemental Figure 4. NSPCs acquire lactate transport machinery but do not lose fatty acid oxidation machinery during differentiation.** Cells were identified in subventricular zone (SVZ), olfactory bulb (OB) and cortex, then laser-capture microdissected. mRNA was isolated, reverse transcribed, and subjected to real-time PCR. Transcripts were normalized to B-actin. Cells located in OB contain similar high levels of transcripts for the fatty acid oxidation enzymes Long-Chain Acyl CoA Dehydrogenase (ACADL, A) and Carnitine Palmitoyl Transferase I (CPT1, B) compared with cells from SVZ, while cortical cells have significantly lower levels of these transcripts compared with SVZ. Both cortical cells and OB cells contain significantly higher levels of transcripts for Monocarboxylate Transporter 2 (MCT2) compared with SVZ cells (C). In a separate experiment, cultured adult NSPCs were collected over 24 hours of serum exposure. This timeframe is sufficient to cause morphological (D-G) and immunohistochemical (H-K) features of astrocytic differentiation. No changes in the quantity of transcripts for ACADL or CPT1 occurred during this time period, although MCT2 transcripts increased significantly at 12 hrs and 24 hrs of serum exposure compared with no serum exposure (L). Scale bars represent 20 μm.

**
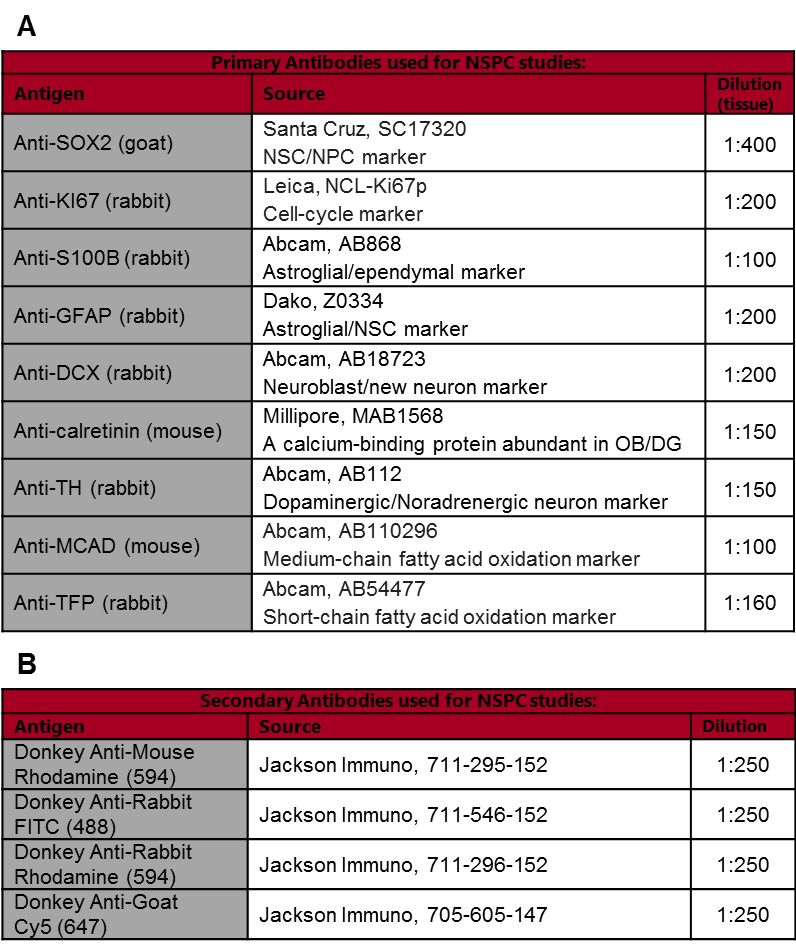
**

**Supplemental Table 1. Antibodies used for immunohistochemistry.** Primary antibodies that were used for immunohistochemical assays in mouse brain tissue for this study are described in (A) and secondary antibodies conjugated to fluorophores that were used to visualise protein expression for this study are described in (B).

**
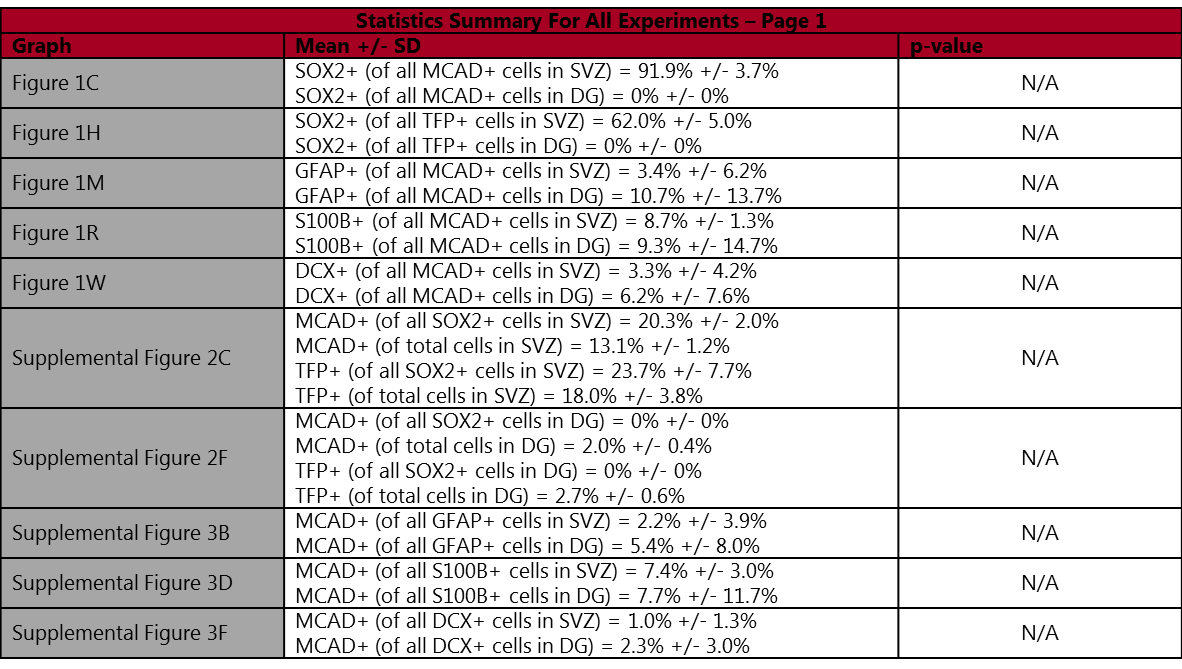
**

**Supplemental Table 2. Page 1. Means, standard errors, and p-values for all experiments in this study.**

**
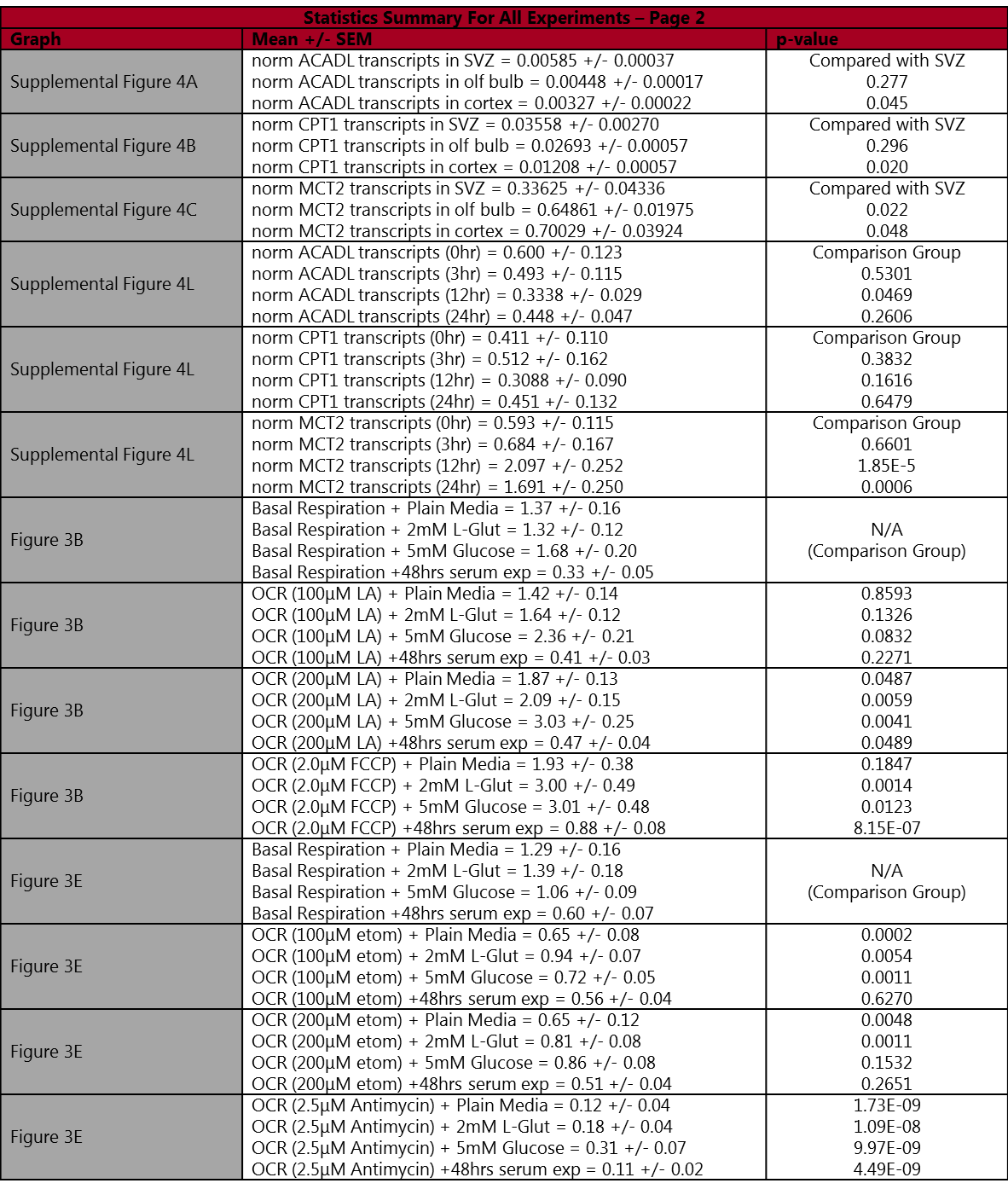
**

**Supplemental Table 2. Page 2. Means, standard errors, and p-values for all experiments in this study.**

**
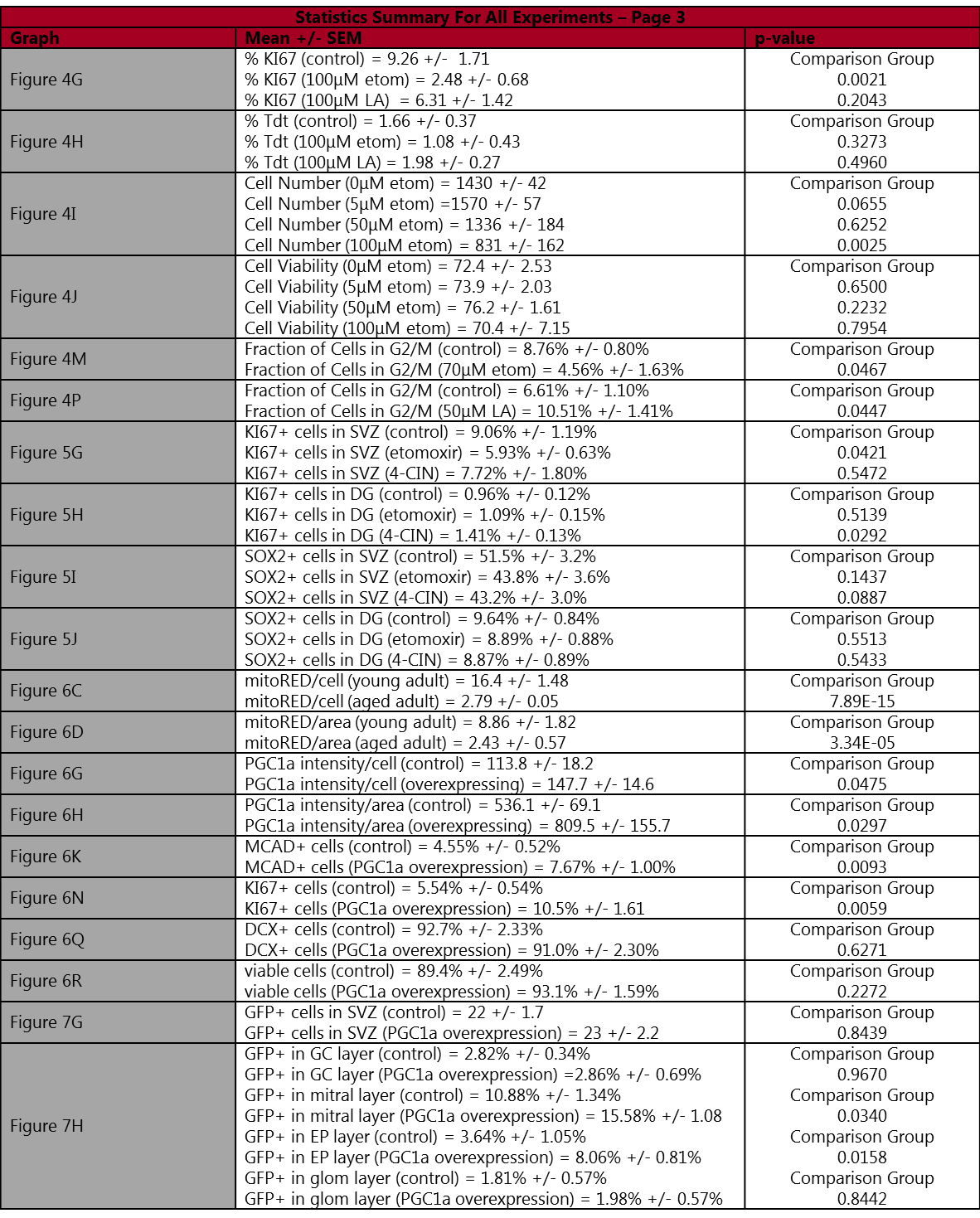
**

**Supplemental Table 2. Page 3. Means, standard errors, and p-values for all experiments in this study.**
